# Trajectories of intimacy and conflict with mothers and fathers from adolescence to adulthood

**DOI:** 10.1177/01650254251364822

**Published:** 2025-08-28

**Authors:** Chloé Charest-St-Onge, François Poulin

**Affiliations:** 1 Université du Québec à Montréal, Canada

**Keywords:** Relationship quality, mother, father, emerging adulthood, trajectories

## Abstract

The quality of mother–child and father–child relationships evolves during emerging adulthood. Given the diverse life paths of young adults, the positive (intimacy) and negative (conflict) dimensions underpinning these relationships may change heterogeneously. This study aimed to identify trajectories of intimacy and conflict with mothers and fathers from ages 16 to 30, explore the overlap between mothers’ and fathers’ trajectories, and examine whether child characteristics and family characteristics predict membership in these trajectories. Data were drawn from a longitudinal study initiated with 390 participants recruited from sixth-grade classes (58% girls). Intimacy and conflicts were measured at ages 16, 17, 18, 19, 20, 21, 22, 25, and 30, and antecedents were measured at ages 12, 13, and 15. Latent class growth analyses revealed three trajectories of intimacy for mothers: (1) low-stable (30%), (2) moderate-quadratic (40%), and (3) high-quadratic (30%); and for fathers: (1) low-stable (44%), (2) moderate-quadratic (44%), and (3) high-quadratic (12%). Two conflict trajectories were identified for mothers: (1) high-linear (18%) and (2) low-quadratic (82%), and for fathers: (1) high-stable (15%) and (2) low-quadratic (85%). Child gender and socioeconomic status predicted mother-intimacy trajectories, while family cohesion and children’s depressive symptoms predicted intimacy and conflict trajectories with both parents, respectively.

## Introduction

The development of relationship quality with mothers and fathers has been the subject of several studies in childhood and adolescence (Kirby & Hoang, 2018), but less in emerging adulthood (EA). This period, extending from 18 to 29 years, is marked by instability, identity exploration, and multiple transitions, such as leaving the parental home and entering the workforce (Arnett, 2015). According to individuation theory, emerging adults continue to gain autonomy through these transitions, leading to adjustments in their relationships with their parents (Buhl, 2008; Koepke & Denissen, 2012). Thus, the positive (e.g., intimacy) and negative (e.g., conflict) dimensions underlying the quality of these relationships may continue to evolve beyond adolescence. Furthermore, certain characteristics of the child and family, present from early adolescence, could be associated with the development of these dimensions (Padilla-Walker & Nelson, 2019). This study aims to describe changes in intimacy and conflict with mothers and fathers from adolescence to adulthood (ages 16–30), explore the links between mothers’ and fathers’ developmental trajectories, and examine predictors.

## Parent–Child Relationship Quality: A Multidimensional Concept

Based on social exchange theory, the quality of parent–child relationships involves both positive and negative dimensions that can coexist (Rook, 1984). Researchers have highlighted the need to assess these two dimensions separately to understand better their development and respective links with children’s adjustment (Lindell & Campione-Barr, 2017; Padilla-Walker & Nelson, 2019; Trentacosta et al., 2011). Previous studies used intimacy and conflict as indicators of these dimensions (e.g., Brouillard et al., 2019). Intimacy is defined as sharing thoughts and feelings with another person (Buhrmester & Furman, 1987), with self-disclosure being an important component (Dubé, 2006). Conflicts are defined as negative interactions or quarrels associated with a generally negative perception of the relationship (Furman & Buhrmester, 2009).

## The Quality of Parent–Child Relationships From Adolescence to Adulthood

Adolescence is marked by biological, cognitive, and social changes, resulting in a growing desire for autonomy (Ralph, 2018). This increase in autonomy extends into EA (ages 18–29), a developmental period characterized by identity exploration and instability (Arnett, 2015). According to social needs theory, friends and romantic partners become increasingly significant in the social sphere of adolescents and emerging adults. Adolescents’ need for autonomy drives them to invest more in friendships, while their sexual needs push them to seek out romantic partners (Weiss, 1974). In response to these changes, the relationship with parents is realigned; it gradually shifts from a vertical one, where parents have more power, to a horizontal one, where interactions are symmetrical and reciprocal (Branje, 2018; Laursen & Collins, 2009).

Moreover, although individuation begins in the early years of life, a second individuation occurs during adolescence (Blos, 1967). This process manifests through decreased idealization and imitation of parents, recognition of their private lives, and increased independence from them. This individuation continues during EA, mainly through transitions such as leaving the parental home, which often come with additional responsibilities and increased independence from parents (Buhl, 2008; Koepke & Denissen, 2012). These transitions can contribute to renegotiating family roles and a more egalitarian relationship with parents. Importantly, individuation does not imply the end of the relationship with parents but rather the achievement of autonomy and the assertion of individuality within a relationship that remains strong. Thus, to foster a healthy individuation process, it is crucial for both the parents and the child to strike a balance between the autonomy gained and the maintenance of a close relationship. Therefore, while intimacy may initially decline slightly, it will likely remain relatively high throughout the individuation process. Regarding conflicts, they can act as a mechanism to steer the relationship’s development toward a more desirable state (Branje, 2018; Buhl, 2008). A healthy individuation process, leading to the desired balance between autonomy and closeness, might be associated with reduced levels of conflict once the desired state of the relationship is reached.

## Development of Intimacy and Conflict With Parents: Findings From Longitudinal Studies

Several studies used latent growth modeling (LGM) to examine the average developmental trend of intimacy and conflict with parents. Overall, they indicate that intimacy temporarily declines during adolescence before stabilizing at the onset of EA, while conflicts decrease in late adolescence and early EA (Fang et al., 2022; Parra et al., 2015). These studies suggest that parent-child relationship quality improves at the end of adolescence and at the beginning of EA, which is consistent with individuation theory.

While these findings provide valuable insights, LGM assumes that the same latent curve can adequately describe all participants (Jung & Wickrama, 2008). This approach ignores the possible existence of subgroups that may follow distinct developmental trajectories, limiting its ability to capture heterogeneity in certain populations, such as emerging adults. Indeed, EA is characterized by heterogeneity in life paths (Arnett, 2015). For example, some emerging adults pursue extended post-secondary studies and maintain a certain dependence on their parents, while others quickly enter the workforce. This heterogeneity may result in the quality of relationships with parents evolving differently for each individual. To better reflect these individual differences, person-centered approaches, such as latent class growth analysis (LCGA), growth mixture model (GMM) and group-based trajectory model (GBTM), are considered more suitable (Jung & Wickrama, 2008; Nagin & Odgers, 2010). Considering heterogeneity, these approaches can capture inter-individual differences in intra-individual change over time. This is essential for identifying potentially higher-risk trajectories.

Studies have used person-centered approaches to investigate parent–adolescent relationships. While Brouillard et al. (2019) identified different warmth and conflict trajectories between ages 13 and 17, they found that most adolescents were classified in high-quality relationship trajectories with both parents. In line with these findings, Yu (2019) identified different conflict trajectories from ages 13 to 19 but found that most participants followed a low-conflict trajectory. Only two studies examined heterogeneity during EA. Doty and Mortimer (2018) measured intimacy with mothers six times between the ages of 15 and 38. LCGA revealed three trajectories: (1) low with quadratic increase (10.8% of the sample), (2) high with quadratic increase (79.4%), and (3) moderate with quadratic decrease (12.9%). Nelson et al. (2015) measured conflicts with parents three times over a year and a half among individuals aged 20.6 on average. LCGA revealed four trajectories: (1) low-stability (65%), (2) linear increase (10%), (3) large linear decrease (14%), and (4) moderate linear decrease (11%). As observed in adolescent studies, most emerging adults experienced trajectories of higher intimacy and lower conflict.

Overall, these studies confirm that the relationship with parents generally improves toward the end of adolescence and suggest that most adolescents and emerging adults experience a positive relationship. However, studies using person-centered approaches show that other patterns of change may exist and present more challenges. Doty and Mortimer (2018) found that individuals in trajectories marked by lower intimacy tended to experience more depressive moods than those in the high intimacy trajectory. Given the different trajectories found in these studies and the theoretical heterogeneity that characterizes EA, person-centered approaches are particularly relevant for examining the development of parent-EA relationships.

## The Importance of Examining Mothers and Fathers Separately and in Conjunction

Mothers and fathers differ in how they engage and behave with their children (Fagan et al., 2014). According to social role theory, these differences are explained by the persistence of traditional definitions of maternal and paternal roles over time (Wood & Eagly, 2012), resulting in parental stereotypes that contribute to the socialization of mothers and fathers (Anderson et al., 2023). Studies suggest that the relationship quality with mothers improves more during EA than with fathers, which remains stable (e.g., Lindell & Campione-Barr, 2017; Memmott-Elison et al., 2021). However, Fang et al. (2021) observed higher intimacy and conflict with mothers at age 17 and a greater decline in contact and conflict with fathers between 17 and 22. Still, none of these studies considered the heterogeneity characterizing EA. During adolescence, Brouillard et al. (2019) found trajectories that were unique to one parent: a low-intimacy trajectory appeared only for fathers, while a high-conflict trajectory was found only for mothers, suggesting that adolescents are likelier to experience higher conflict with their mother, and lower intimacy with their father. Overall, these findings highlight the importance of distinguishing between mother–child and father–child relationships to gain a deeper understanding of their development.

However, according to family system theory, mothers and fathers are interconnected within the family unit (Johnson & Ray, 2016). Consequently, changes in intimacy and conflict with one parent are likely associated with changes in the relationship with the other parent. Xie et al. (2021) found that a greater decline in mother–child intimacy was associated with a greater decline in father-child intimacy from mid-childhood to adolescence, while increased conflict with mothers was linked to increased conflict with fathers. These results suggest a spillover effect. These findings suggest the relevance of exploring how changes in intimacy (or conflict) with one parent may be associated with changes in intimacy (or conflict) with the other parent. In summary, studying mothers and fathers separately and in conjunction is needed to capture the dynamics between them.

## Factors Associated With Trajectory Membership

Beyond describing the development of mother–child and father–child relationships, examining the sources of change within these relationships can provide insight into promoting positive development and preventing deterioration. Thus, parent-child relationships are shaped by child- and family-driven factors (e.g., Oliveira et al., 2020; Trentacosta et al., 2011). While early research focused on family-driven predictors, it is now well established that child characteristics also shape these relationships (Laursen & Collins, 2009). This idea is supported by the life course perspective’s principle of linked lives, along with the family systems approach, which both suggest that changes in family relationships over time can be associated with individual characteristics of family members, including the child, as well as broader family characteristics as a system (Baptist & Hamon, 2022; Charruault, 2020). Indeed, both child-driven (gender, externalization/internalization problems) and family-driven (socioeconomic status, family structure, family cohesion) antecedents will be considered.

### Child’s Gender

Differential socialization experiences for boys and girls may lead to differences in the quality of their relationships with their mothers and fathers, even up to EA (Brown et al., 2020; Cislaghi & Heise, 2020). Some longitudinal studies covering adolescence and/or EA support this idea (e.g., (Brouillard et al., 2019; Parra et al., 2015), while others do not (e.g., Nelson et al., 2015; Yu, 2019). Thus, the role of the child’s gender in the development of intimacy and conflicts with mothers and fathers remains unclear.

### Externalizing and Internalizing Problems

Regarding externalizing problems, Padilla-Walker et al. (2018) observed that adolescents with higher levels of delinquency at age 12 disclosed less to their parents between 12 and 18. Yu (2019) reported that more aggressive adolescents at age 13 were more likely to be assigned to a high (versus moderate) trajectory of conflicts with parents from ages 13 to 19. For internalizing problems, the interpersonal theory of depression (Coyne, 1976) suggests that depressive symptoms can elicit negative reactions from one’s environment and deteriorate the quality of relationships. Furthermore, Brouillard et al. (2019) found that depressive symptoms between ages 13 and 17 were associated with a higher likelihood of being assigned to lower warmth trajectories between ages 13 and 17 and higher conflict trajectories with both parents. It is reasonable to believe that these links between externalizing/internalizing problems and trajectories of intimacy and conflicts with mothers and fathers may also be observable during EA.

### Socioeconomic Status and Family Structure

According to the family stress model, low-income and/or parental separation concerns could make parents less available, less responsive, and more hostile (Masarik & Conger, 2017; Zemp & Bodenmann, 2018). Over time, this could lead to a more distant and conflictual relationship with their child. Doty and Mortimer (2018) found that lower family income was associated with a higher likelihood of being assigned to low and moderate intimacy trajectories (vs high). Fang et al. (2022) reported that lower income was linked to a greater decline in parental warmth between ages 11 and 18 and fewer contacts at age 22. In addition, children who lived with both parents at age 11 reported more contact and intimacy with them at age 22.

### Family Cohesion

Family cohesion is defined as the emotional bond among family members (Olson, 2000). Low cohesion is associated with disengaged relationships and little support between family members. Conversely, high cohesion is linked to better communication between parents and children (Farrell & Barnes, 1993). Thus, children who grow up in such an environment may feel closer to their parents and experience fewer conflicts during EA. Research indicates that earlier family interactions may contribute to the future quality of parent-child relationships (e.g., Laursen et al., 2010). This continuity can be explained by social learning theory, which posits that interaction styles learned in childhood persist throughout life (Whitbeck et al., 1994).

## The Present Study

This study aims to address three gaps identified in the current literature. First, besides Doty and Mortimer (2018), no other studies examined changes in parent-child relationship quality throughout the entire EA period, which would be advised as this relationship theoretically continues to evolve. Second, few studies consider the heterogeneity that characterizes the EA period by identifying distinct trajectories of intimacy or conflict (Doty and Mortimer, 2018; Nelson et al., 2015). Third, few studies include both parents, and only one examines different trajectories of change for mothers and fathers, although this is limited to adolescence (Brouillard et al., 2019).

This study has three objectives. The first is to examine changes in intimacy and conflict, respectively, with mothers and fathers from mid-adolescence to the end of EA while considering the heterogeneity of this period. These variables were measured at nine time points between ages 16 and 30. It is expected that distinct trajectories will emerge for each relational contexts examined (mother intimacy, father intimacy, mother conflict, father conflict). For mother intimacy, at least three trajectories are expected: (1) low intimacy increasing, (2) high intimacy increasing, and (3) moderate intimacy decreasing (Doty & Mortimer, 2018). No specific hypothesis is formulated for father intimacy, as Doty and Mortimer’s (2018) study did not include fathers. No hypothesis is made regarding the number of trajectories for conflicts with mothers and fathers, as no study has measured conflicts with parents from the start to the end of EA. However, based on general trends, at least two trajectories are expected to emerge, one showing a decrease in conflicts (e.g., Fang et al., 2021; Parra et al., 2015). Moreover, it is expected that most individuals will experience a healthy individuation process, leading them to fall into medium to high intimacy trajectories and into a low-conflict trajectory.

The second objective is to examine how changes in intimacy (or conflict) with one parent can be associated with changes in intimacy (or conflict) with the other parent. Based on the study by Xie et al. (2021), it is expected that individuals who experience less intimacy (or conflict) with mothers will also experience less intimacy (or conflict) with fathers, and vice versa.

The third objective is to examine factors that may lead to membership in these trajectories. Child characteristics (gender, externalizing and internalizing problems) and family characteristics (socioeconomic status, family structure, family cohesion) measured in early adolescence are considered. No specific hypothesis is made regarding the child’s gender due to the contradictory results of previous studies. Based on theories and empirical studies (e.g., Brouillard et al., 2019; Doty & Mortimer, 2018; Fang et al., 2022; Laursen et al., 2010; Yu, 2019), it is expected that higher levels of externalizing/internalizing problems, lower socioeconomic status, separated parents, and lower family cohesion will be associated with membership in low-quality relational trajectories, characterized by lower intimacy and higher conflict.

## Methodology

### Participants

This longitudinal study began in 2001 with 390 sixth-grade students (58% female; *M*age = 12.38; *SD* = 0.42) recruited from eight elementary schools in a large city north of Montreal, Quebec. The majority (90%) of the participants were white, with the remainder being Black (3%), Hispanic (3%), Arab (3%), and Asian (1%). Most came from middle-class families with an average family income between $45,000 and $55,000 at the start of the study. Participants were assessed repeatedly up to the age of 30. The sample used for this study included participants who completed relationship quality measures with the mother or father at least once between ages 16 and 30. In total, 368 participants (59% female) met this criterion. They did not differ from those not included in terms of gender but were more likely to come from intact families (χ^2^ = 2.87, *p* < .001) with higher income (*t*(288) = −3.34, *p* < .001).

### Procedures

At the start of the study, parents filled out a questionnaire their child brought home. At ages 12, 13, 15, 16, and 17, children completed questionnaires in class under the supervision of research assistants. Research assistants visited participants’ homes at ages 18, 19, 20, 21, 22, and 25 to administer the questionnaires. At age 30, questionnaires were completed online via the LimeSurvey platform. Parental consent was obtained each year until age 18, after which participants consented for themselves. Participants received small gifts (e.g., stickers, pens) in the early years and financial compensation in later years. The study was approved by the Université du Québec à Montréal Research Ethics Committee (approval number: 2022-4748).

### Measures

#### Relationship Quality With Mother and Father at Ages 16, 17, 18, 19, 20, 21, 22, 25, and 30

Relationship quality was measured using the intimate disclosure and conflict subscales of the Network of Relationship Inventory (Furman & Buhrmester, 2009), completed by the child. This instrument is designed for use in various relational contexts, including relationships with the mother and father. Participants answered statements referring to their mother and then their father. Each subscale consisted of three statements. An example of an intimacy statement is “How much do you talk about everything with your mother/father?” and for conflict, “How often do you and your mother/father disagree and quarrel with each other?” Responses were on a 5-point Likert-type scale ranging from 1 (*Little or none*) to 5 (*The most*). The mean score of the three statements was calculated for each subscale. In this sample, Cronbach’s alphas ranged from .79 to .90 for intimacy and .82 to .91 for conflict across measurement points.

#### Externalizing Problems (Ages 12–13)

Externalizing problems were measured using a 16-item scale combining the Metzler et al. (1998) antisocial behavior scale and items from a scale developed by Janosz and Bouthillier (2007). These items covered problems occurring at home, in class, or with peers (e.g., lying to parents, hitting or threatening other students, stealing). Participants responded on a 6-point Likert-type scale from 1 (never) to 6 (more than 10 times) to indicate how often they exhibited the behavior over the past month. The score was calculated by averaging the 16 items. In this sample, Cronbach’s alphas were .83 at age 12 and .82 at age 13. A final score was obtained by averaging scores from ages 12 and 13 (*r* = .60).

#### Internalizing Problems (Ages 12–13)

Internalizing problems were measured using the Child Depression Inventory by Kovacs et al. (1981). This instrument includes 27 sets of statements assessing the severity of affective, behavioral, and cognitive depressive symptoms in children. The set of statements related to suicidal ideation was excluded for ethical reasons. Participants chose the one that best described how they had felt over the past 2 weeks for each set of statements. For example, they could select: “I have trouble sleeping every night,” “I have trouble sleeping many nights,” or “I sleep pretty well.” The score for depressive symptoms was calculated by summing the scores of the 26 sets of statements. In this sample, Cronbach’s alphas were .85 at age 12 and .82 at age 13. A final score was obtained by averaging the scores from ages 12 and 13 (*r* = .59).

#### Socioeconomic Status (Age 12)

Parents reported their total annual family income before tax using a 13-option response scale ranging from “Less than $5,000” to “$60,000 or more.”

#### Family Structure (Age 15)

Participants answered the question “Which best describes your current living situation?” by choosing one of six options, such as: “I live with both parents” and “I live one week with my mother and one week with my father.” Responses were recoded into two levels: 1 = lives with both parents, 2 = does not live with both parents.

#### Family Cohesion (Ages 12–13)

Family cohesion was measured using the positive family relationships subscale from a questionnaire assessing various aspects of the parent-child relationship (Metzler et al., 1998). It comprises six statements addressing emotional bonds and positive functioning among family members. An example is “Family members help each other.” Participants responded on a 5-point Likert-type scale from 1 (*not at all true*) to 5 (*completely true*). The score was calculated by averaging the six statements. In this sample, Cronbach’s alphas were .88 at age 12 and .91 at age 13. A final score was obtained by averaging the scores from ages 12 and 13 (*r* = .62).

### Analysis Plan

To address the first objective, latent growth modeling (LGM) was conducted for each relational context (mother intimacy, father intimacy, mother conflict, father conflict) to determine the form of change (e.g., linear vs quadratic) across the sample (Little, 2013). Increasingly complex models were tested to identify a model with adequate fit indices. Initially, only the intercept (i) was included, followed by the addition of a linear slope (i and s), and finally, a quadratic slope (i, s, and q). Model fit indices used included the root mean square error of approximation (RMSEA), the Bentler comparative fit index (CFI), the Tucker–Lewis index (TLI), and the standardized root mean square residual (SRMR). A model fits well if RMSEA is below .08, CFI and TLI are .95 or higher, and SRMR is .10 or below (Kline, 2016). The variance was estimated for all latent factors (i, s, and q), and significant variances suggested heterogeneity and the need to proceed with latent class growth analyses (LCGA).

LCGA identifies subgroups of individuals with distinct patterns of change within the sample (Jung & Wickrama, 2008). Models with 2, 3, 4, and 5 classes were tested. The optimal number of classes was determined based on model fit indices: the Bayesian information criterion (BIC), the sample-size adjusted BIC (SSABIC), the Akaike information criterion (AIC), BLRT and LMR-LRT tests, entropy, and the posterior probability of correct classification. Lower AIC, BIC, and SSABIC scores indicate a better model fit (Jung & Wickrama, 2008). A significant LRT test (*p* < .05) indicates that the tested model is better than a model with one fewer class (Nylund et al., 2007), and entropy of at least .80 indicates well-determined classes (Clark & Muthén, 2009). A posterior probability of 1 means 100% of individuals were well classified, increasing confidence in the classification (Jung & Wickrama, 2008). The number of individuals per class was also considered. Analyses were conducted using Mplus software, employing the full information maximum likelihood estimation method for handling missing data (Baraldi & Enders, 2010).

Joint trajectories were conducted to meet the second objective (Nagin & Odgers, 2010). This approach enables examining the links between the developmental trajectories of two distinct variables, which are theoretically related and evolve simultaneously. It calculates the probability of belonging to a specific intimacy (or conflict) trajectory with one parent based on membership in a specific intimacy (or conflict) trajectory with the other. Thus, after determining the optimal number of trajectories for each relational context, two additional analyses were conducted: (1) joint trajectories of intimacy with mothers and fathers in the same model and (2) joint trajectories of conflict. These joint models were used for subsequent analyses regarding the predictors.

Four trajectory membership variables were created to address the third objective by calculating the most probable class membership for each individual, for each relational context, using the joint models. These variables were then exported into SPSS software. Multiple imputations were performed for independent variables that had more missing data. Binary logistic regressions (if two trajectories) and multinomial (if more than two trajectories) were conducted with trajectory membership as dependent variables and predictors as independent variables. All predictors were tested simultaneously. For each model, the least problematic trajectory was used as the reference trajectory (the trajectories showing the most intimacy and the least conflict).

## Results

The correlations between relationship quality variables and predictors and the means and standard deviations of quality variables are presented in Supplemental Material 1 Table 1.

### Objective 1: Trajectories of Intimacy and Conflicts With Mothers and Fathers From Ages 16 to 30

Results of LGM analyses indicated better models fit when the intercept, linear slope, and quadratic slope were calculated (see Supplementary 1 Table 2). The quadratic model was thus retained for each relational context. Furthermore, the variances of all latent factors were significant (mother intimacy: I = 1.10, *p* = .000; S = 4.99, *p* *=* .000; Q = 2.06, *p* *=* .000; father intimacy: I = 0.62, *p* = .000; S = 4.74, *p* *=* .000; Q = 1.84, *p* *=* .000; mother conflict: I = 0.60, *p* = .000; S = 2.36, *p* *=* .000; Q = 0.92, *p* *=* .000; father conflict: I = 0.51, *p* = .000; S = 2.28, *p* *=* .000; Q = 0.77, *p* *=* .000) confirming the presence of heterogeneity within the sample and the relevance of conducting latent class analyses.

Results of LCGA analyses are presented in the Supplemental Material 1 Table 3. The models were adjusted to include different types of trajectories (e.g., quadratic and linear trajectories within the same model) when non-significant polynomial terms suggested their relevance.

#### Mother Intimacy

**Figure 1.**
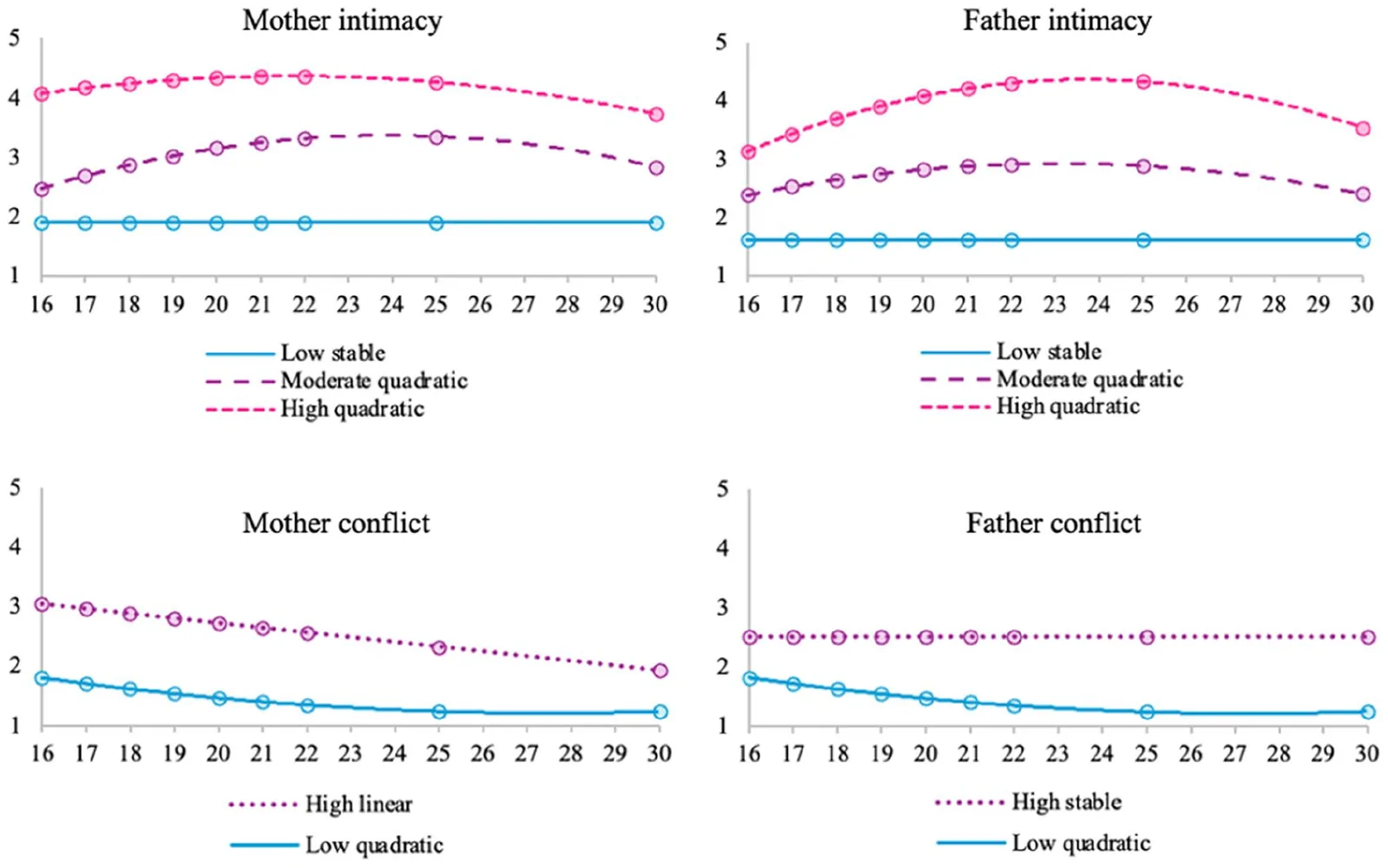
Trajectories of intimacy and conflict from ages 16 to 30. Note. Age is on the x-axis. Intimacy is on the y-axis for Mother and Father intimacy. Conflict is on the y-axis for Mother and Father conflict. N for mothers = 368. N for fathers = 361.

Although the LRT tests were significant up to the five-trajectory model, entropy and classification probability decreased from the four-trajectory model onward. The three-trajectory model was thus retained (see Figure 1): 30% of the sample was assigned to the low and stable intimacy group (I = 1.90, *p* = .000); 40% to the moderate intimacy group, which increases and stabilizes quadratically (I = 2.48, *p* = .000, S = 2.25, *p* = .000, Q = -1.43, *p* = .000) and 30% to the high intimacy group, which increases and stabilizes quadratically (I = 4.07, *p* = .000, S = 1.03, *p* = .000, Q = -.91, *p* = .000).

#### Father Intimacy

The LRT tests for the three-trajectory model were significant, and the fit indices remained satisfactory but deteriorated from the four-trajectory model onward. The three-trajectory model was thus retained (see Figure 1): 44% of the sample was assigned to the low and stable intimacy group (I = 1.63, *p* = .000); 44% to the moderate intimacy group, which increases and stabilizes quadratically (I = 2.39, *p* = .000, S = 1.53, *p* = .000, Q = −1.09, *p* = .000) and 12% to the high intimacy group, which increases and stabilizes quadratically (I = 3.14, *p* = .000, S = 3.19, *p* = .000, Q = −2.07, *p* = .000).

#### Mother Conflict

The LRT tests for the two-class model were significant, and the fit indices were satisfactory. The three-trajectory model’s LMR LRT test was insignificant, and the indices deteriorated from the four-trajectory model onward. In addition, the smallest class in models with more than two trajectories comprised only 1% to 2% of the sample. The two-class model was therefore retained (see Figure 1): 18% of the sample was assigned to the high-conflict group, which decreases linearly (I = 3.05, *p* = .000, S = −0.81, *p* = .000) and 82% to the low-conflict group, which decreases and stabilizes quadratically (I = 1.80, *p* = .000, S = −1.04, *p* = .000, Q = 0.45, *p* = .000).

#### Father Conflict

The three-class model presented good fit indices and significant LRT tests, but its smallest class comprised 4% of the sample (*n* = 13). The LRT tests for the two-class model were significant, and the fit indices were satisfactory. The two-class model was thus retained (see Figure 1): 15% of the sample was assigned to the high and stable conflict group (I = 2.50, *p* = .000) and 85% to the low-conflict group, which decreases and stabilizes quadratically (I = 1.82, *p* = .000, S = −1.08, *p* = .000, Q = 0.48, *p* = .003).

### Objective 2: Joint Trajectories Linking Mothers and Fathers

Tables 1 and 2 present the probabilities of belonging to the different intimacy and conflict trajectories for one parent, according to the trajectory membership for the other. Some results suggest that the quality of the relationship maintained with one parent was similar to that experienced with the other. For instance, participants classified in the low-intimacy trajectory with their mother had a 76% probability of also being in the low-intimacy trajectory with their father. Similarly, those assigned to a low-conflict trajectory with their mother or father had a high probability of experiencing low conflicts with the other (87% and 85% odds, respectively). However, other results suggest that this pattern of similarities was not always present. For example, participants in the high intimacy trajectory with the mother had only a 24% chance to show high intimacy with the father. For conflict, participants assigned to the high-conflict trajectory with the mother or father had a 35% and 38% chance, respectively, of being also assigned to a high-conflict trajectory with the other parent. Thus, for the majority, a high level of conflict with one parent was not associated with a high level of conflict with the other. While some aspects of relationship quality with both parents aligned, exceptions are noted. Since the findings suggest interdependence in the development of mother–child and father–child relationships, additional exploratory analyses were conducted using a parallel-process LCGA procedure. This approach allows for the simultaneous analysis of mother and father intimacy development within a single LCGA. The same analysis was conducted for mother conflict and father conflict. These analyses and the corresponding results are presented in supplementary material 2. Overall, the results provide support for some of the findings obtained from the joint trajectories, while also revealing similar trajectories to those identified by the univariate LCGAs presented above.

**Table 1.** Co-occurrence of Mother-Intimacy and Father-Intimacy Trajectories.

	Probability of father intimacy group conditional on mother intimacy group		
	Father low	Father moderate	Father high
Mother low	0.755	0.206	0.039
Mother moderate	0.297	0.613	0.090
Mother high	0.302	0.461	0.237
	Probability of mother intimacy group conditional on father intimacy group		
	Mother low	Mother moderate	Mother high
Father low	0.522	0.272	0.206
Father moderate	0.140	0.552	0.309
Father high	0.100	0.305	0.595

*Note. N* = 361.

**Table 2.** Co-occurrence of Mother-Conflict and Father-Conflict Trajectories.

	Probability of father conflict group conditional on mother conflict group	
	Father low	Father high
Mother low	0.872	0.128
Mother high	0.651	0.349
	Probability of mother conflict group conditional on father conflict group	
	Mother low	Mother high
Father low	0.850	0.150
Father high	0.607	0.393

*Note. N* = 361.

### Objective 3: Predictors of Trajectories

The results are presented in Table 3. For intimacy with the mother, the child’s gender, socioeconomic status, and family cohesion predicted trajectory membership. Specifically, men were more likely to be assigned to low and moderate intimacy trajectories compared to the high intimacy trajectory (*p* < .001 and *p* < .001 respectively). Individuals with higher family socioeconomic status were more likely to be assigned to the low-intimacy trajectory compared to the high trajectory (*p* = .015). In addition, individuals with higher family cohesion were less likely to be assigned to low and moderate intimacy trajectories compared to the high trajectory (*p* < .001 and *p* < .001 respectively). For intimacy with the father, family cohesion predicted trajectory membership. Individuals with higher family cohesion were less likely to be assigned to the low-intimacy trajectory compared to the high trajectory (*p* = .022).

**Table 3.** Predictors of Intimacy and Conflict Trajectories.

	Mother intimacy: low versus high			Mother intimacy: moderate versus high			Father intimacy: low versus high		
Predictors	Estimate	Odds ratio	95% CI	Estimate	Odds ratio	95% CI	Estimate	Odds ratio	95% CI
Gender	1.34***	3.84	[2.04, 7.23]	1.05***	2.86	[1.59, 5.17]	–0.02	0.98	[0.46, 2.13]
Externalizing problems	–0.73	0.48	[0.23, 1.001]	–0.64	0.53	[0.27, 1.04]	–0.73	0.48	[0.18, 1.30]
Internalizing problems	0.04	1.04	[0.98, 1.11]	0.01	1.01	[0.95, 1.06]	0.06	1.06	[0.97, 1.15]
Socioeconomic status	0.13*	1.14	[1.03, 1.27]	0.08	1.08	[0.96, 1.21]	0.09	1.10	[0.96, 1.25]
Family structure	0.66	1.94	[0.97, 3.88]	0.41	1.50	[0.80, 2.84]	0.55	1.74	[0.69, 4.38]
Family cohesion	–0.75***	0.47	[0.31, 0.72]	–0.71***	0.49	[0.33, 0.73]	–0.70*	0.50	[0.28, 0.90]
	Father intimacy: moderate versus high			Mother conflict: high versus low			Father conflict: high versus low		
	Estimate	Odds ratio	95% CI	Estimate	Odds ratio	95% CI	Estimate	Odds ratio	95% CI
Gender	0.31	1.37	[0.64, 2.91]	–0.52	0.60	[0.32, 1.11]	0.03	1.03	[0.55, 1.93]
Externalizing problems	–0.12	0.88	[0.34, 2.28]	–0.59	0.56	[0.27, 1.14]	–0.42	0.66	[0.32, 1.37]
Internalizing problems	0.04	1.04	[0.96, 1.13]	0.06*	1.07	[1.01, 1.12]	0.06*	1.06	[1.004, 1.13]
Socioeconomic status	0.02	1.02	[0.89, 1.16]	0.04	1.04	[0.94, 1.16]	–0.07	0.93	[0.83, 1.05]
Family structure	–0.07	0.94	[0.38, 2.31]	0.16	1.17	[0.57, 2.40]	0.09	1.10	[0.45, 2.70]
Family cohesion	–0.55	0.58	[0.32, 1.04]	–0.34	0.71	[0.49, 1.02]	–0.15	0.86	[0.57, 1.29]

*Note*. N for mothers = 368. N for fathers = 361. For gender, Female is coded as 0, and Male is coded as 1. For the family structure variable, parents together are coded as 1, and parents separated are coded as 2.

*** *p* < .001, *p < .05.

For the conflict trajectories, for both the mother and the father, depressive symptoms predicted trajectory membership. A higher level of depressive symptoms was associated with a greater probability of being assigned to the high-conflict trajectory compared to the low-conflict trajectory (*p* = .022 for mothers and *p* = .036 for fathers).

In summary, the child’s gender and socioeconomic status only predicted membership in mother-intimacy trajectories. Family cohesion and depressive symptoms predicted membership in intimacy and conflict trajectories, respectively, with both parents.

## Discussion

This study aimed to examine changes in intimacy and conflicts with mothers and fathers from ages 16 to 30, explore the links between mothers’ and fathers’ trajectories, and verify whether changes varied according to characteristics specific to the child and the family. For each relational context, distinct trajectories were identified, some of which overlap between the two parents. In addition, several characteristics measured at the beginning of adolescence predicted membership in these trajectories.

### Changes in Intimacy and Conflicts With Mothers and Fathers From Ages 16 to 30

The first objective was to explore changes in intimacy and conflict with mothers and fathers from mid-adolescence to the end of EA. As expected, three trajectories were identified for intimacy with the mother: (1) low-stable, (2) moderate with quadratic increase, and (3) high with quadratic increase. The low-intimacy trajectory remained stable in this study, whereas it increased in Doty and Mortimer’s (2018) study, which inspired the hypotheses. In addition, no decreasing trajectory was identified here, unlike in their study. The periods covered could explain these differences (ages 15 to 38 for Doty and Mortimer). Three trajectories were identified for fathers, matching the forms found for mothers. The low and moderate intimacy quadratic shape suggests an increase in intimacy between 16 and around 25, and a slight decrease until 30. This finding is consistent with the high intimacy quadratic curve found by Doty and Mortimer (2018). The latter increased from age 15 to around age 25 and then decreased slightly until age 38, comprising most of their sample (76%). This decrease in intimacy observed for 70% and 56% of the sample for mothers and fathers, respectively, in the present study suggests that maintaining a balance between closeness and autonomy, in line with individuation process, remains a challenge during this period. Some authors have stated that individuation remains an important issue even in adulthood (e.g., Buhl, 2008). Since individuation takes place mostly through life transitions during EA, this decrease in intimacy may be related to transitions occurring in the late twenties (Buhl, 2008; Koepke & Denissen, 2012). Indeed, many of these still take place at the end of EA. For example, in Quebec, where the study took place, the mother’s average age at the birth of her first child was 29.5 years in 2021 (Charbonneau & St-Amour, 2022). Regarding conflicts with the mother, as expected, two trajectories were identified: (1) high with linear decrease and (2) low with quadratic decrease. Two trajectories were also identified for conflicts with the father, with the distinction that the high one remained stable. Most of these findings align with studies that reported declining parent-child conflicts in late adolescence and the beginning of EA (e.g., (Fang et al., 2021; Parra et al., 2015). However, Nelson et al. (2015) identified four conflict trajectories, and (Yu, 2019) three, while only two emerged in the present study. These studies, however, did not distinguish between mothers and fathers and did not cover the entire EA period.

Three major conclusions can be drawn from the results of the trajectory analyses. First, most participants maintain positive relationships with their parents during EA. In fact, 70% and 56% of them were assigned to the moderate or high intimacy class, while only 18% and 15% were assigned to the high-conflict class with mothers and fathers, respectively. This aligns with other studies showing that emerging adults maintain positive relationships with their parents (e.g., Doty & Mortimer, 2018; Fang et al., 2021). These results also support the hypotheses derived from individuation theory. Indeed, most emerging adults seem to experience a balanced individuation process, maintaining a certain level of intimacy and minimal conflict with their parents. Second, intimacy and conflicts with parents do not evolve in the same way for everyone, supporting our hypothesis. This phenomenon may be explained by the demographic heterogeneity characterizing EA (Arnett, 2015). Since emerging adults have opportunities to explore various future possibilities, their life paths can differ from one another, leading to different changes in the quality of relationships with their parents. These results are consistent with previous studies that reported heterogeneity in the development of these relationships (Doty & Mortimer, 2018; Yu, 2019). Parents continue to play a significant and positive role for most individuals in EA (Lindell & Campione-Barr, 2017; Padilla-Walker & Nelson, 2019). In fact, a positive relationship with parents is associated with different aspects of adaptation during this period (e.g., Memmott-Elison et al., 2021). However, the current findings demonstrate that not all emerging adults have the opportunity to experience these positive relationships and their associated benefits.

Third, although the overall patterns of change were quite similar for mothers and fathers, some differences emerged. For intimacy, the high trajectory starts and remains at a higher level for the mother compared to the father (at age 16, scores of 4.1 vs 3.1). In addition, participant distribution among each trajectory differs: 30% of the sample was assigned to the high intimacy class for the mother, compared to only 12% for the father. These results are consistent with social role theory, which argues that parental stereotypes contribute to parental socialization and perpetuate their differences (Wood & Eagly, 2012). A study by Anderson et al. (2023) highlights the persistence of parental stereotypes in children’s books, showing that fathers continue to be portrayed as less affectionate and involved. Fathers may conform to these stereotypes, being less affectionate with their children, which could impact the level of intimacy shared with them, even into adulthood. This finding also aligns with studies reporting higher intimacy with the mother at the end of adolescence and the start of EA (Fang et al., 2021; Memmott-Elison et al., 2021). The present study shows this difference persists at least until age 30.

For conflict, the high trajectory begins with a higher score for the mother than the father (at age 16, scores of 3.1 vs 2.5). Greater emotional and physical involvement from mothers, as well as their management of daily tasks, could promote more intimacy with the child but also lead to more conflicts (Ciciolla & Luthar, 2019; Tsai et al., 2023). This high trajectory decreases for mothers but remains stable for fathers. This may be due to mothers engaging more in positive conflict resolution (Van Doorn et al., 2011).

### Links Between Mothers and Fathers: Joint Trajectories

The second objective was to examine the links between mothers’ and fathers’ developmental trajectories of intimacy and conflict using joint trajectories. Some findings support the hypothesis that individuals who experience lower intimacy (or conflict) with one parent will also experience lower intimacy (or conflict) with the other. This suggests a tendency for individuals to follow similar trajectories of intimacy or conflict with both parents, indicating the possibility of a spillover effect between the two relationships. Xie et al. (2021) identified this effect between mother–child and father–child relationships during childhood and adolescence. The current findings suggest that it could also be observed in EA. The parallel-process LCGA reported in supplemental material 2 supports these results, identifying classes with similar levels of intimacy with both parents throughout the period, and a class with low conflict with both parents.

However, participants with high conflict with one parent were likelier to experience low conflict with the other, contradicting the spillover effect. These results can be understood through the lens of compensation theory, which offers alternative hypotheses on how relationships with mothers and fathers may be linked (Nelson et al., 2009). It suggests that an individual may compensate for a poor-quality relationship by investing more in another relationship, which may lead to a better quality of the latter. Thus, a person with a more conflictual relationship with one parent may seek to establish a more positive relationship with the other, and parents may also contribute to this process. Mudrick et al. (2023) observed that the mother became more emotionally focused on the child during times of conflict between the father and the child. This theory could explain why an individual who experiences higher conflict or less intimacy with one parent might be more likely to have less conflict or more intimacy with the other parent. The parallel-process LCGA presented in supplemental material 2 also confirms the presence of compensation, identifying a class in which intimacy is high for mothers and low for fathers, although this can also be explained by social role theory. However, no class characterized by high conflict with one parent and low conflict with the other have been identified; in line with compensation theory, this pattern would be likely to emerge in a larger sample.

### Predictors of Intimacy Trajectories

The third objective was to determine whether child and family characteristics predict membership of the different trajectories. Family cohesion at the beginning of adolescence predicted changes in intimacy with both mothers and fathers. As expected, a high level of cohesion increased the likelihood of being part of the high intimacy trajectory. These results illustrate continuity in the parent-child relationship, as proposed by social learning theory (Whitbeck et al., 1994). This theory suggests that interaction patterns learned in childhood and adolescence continue to manifest in relationships later in life. This also aligns with studies showing that earlier positive family relationships predict better-quality relationships during EA (Fang et al., 2022; Parra et al., 2015). Thus, our results suggest that a more functional family environment in early adolescence, characterized by higher family cohesion, fosters the maintenance of closeness with both mothers and fathers during EA.

Child gender and family socioeconomic status predicted membership in intimacy trajectories only for the mother. Women were likelier than men to be assigned to the high trajectory (vs low and moderate). This result could be explained by the differential socialization of women and men, which encourages them to follow distinct gender norms (Brown et al., 2020; Cislaghi & Heise, 2020). Gender stereotypes are observed in various media, where the characteristics of female characters correspond to empathy and sensitivity (Brown et al., 2020). Therefore, women may develop more intimate relationships with their surroundings, specifically with their mother, who was also socialized as a woman. This result is consistent with studies reporting that girls feel closer to their parents and/or mothers (e.g., Fang et al., 2022). Child gender does not predict intimacy with the father or conflicts with either parent. These findings align with those of previous studies. Brouillard et al. (2019) observed that child gender predicted intimacy trajectories only with the mother. Other studies showed that gender did not predict conflict trajectories (Nelson et al., 2015; Yu, 2019). According to Nelson et al. (2015), gender differences in parent-child conflicts may lie more in the topics of disagreement, an aspect not considered in this study.

Contrary to the proposed hypothesis, participants from higher-income families were less likely to be on a high intimacy trajectory with the mother. This result may be explained by high-income parents tending to have more demanding jobs, being preoccupied with work, and being less available (Luthar & Latendresse, 2005). High work demands may reduce parental availability and thus impact intimacy with their children. Greater occupational stress for women (Herrero et al., 2012) may explain why this result is specific to mothers. Despite the goal of equality, women continue to be seen as responsible for household and family tasks, which may increase the overload experienced and further reduce their availability (Aarntzen et al., 2023). Socioeconomic status was not associated with intimacy with the father or conflicts. Since our sample came from the middle class, the effects of income on these variables might only appear under greater economic stress.

### Predictors of Conflict Trajectories

Depressive symptoms in early adolescence predicted membership in conflict trajectories with both mothers and fathers. As expected, higher symptoms were associated with more conflicts throughout EA. This result aligns with research indicating that depressive symptoms predict higher levels of conflict with parents during adolescence (Brouillard et al., 2019; Yu, 2019). Our study shows that this effect persists throughout EA. Several explanations can be proposed. First, Coyne’s (1976) interpersonal theory of depression suggests that certain manifestations of depression (e.g., irritability, apathy, and negativity) can elicit negative reactions from others (Segrin, 2011). Initially, people around the individual respond to depressive symptoms with reassurance and empathy, but when negative interactions persist, they may begin to react more negatively. These negative reactions could lead to more conflicts between the child and both parents. Second, as mentioned earlier, parent-child relationships have a degree of developmental continuity. The conflictual dynamics established in adolescence, related to the child’s depressive symptoms, may persist over the years. Third, depressive symptoms are often associated with a deficit in social skills (Segrin, 2011). Adolescence is an important period for developing social skills (Ralph, 2018). Therefore, depressive symptoms during this period may impede their development, consequently raising the risk of maintaining more conflictual relationships in the long term.

### Non-significant Predictors

Contrary to the proposed hypotheses, family structure and externalizing problems in early adolescence were unrelated to intimacy and conflict trajectories. For family structure, the effects of separation may have diminished over time; although parental separation is stressful in the short term, most families do not report negative long-term effects (Kelly & Emery, 2003). Furthermore, some authors suggest that the negative effects of separation are more related to parental conflict than to the separation itself (Feeney & Monin, 2018). For externalizing problems, given the low levels reported by our sample (mean of 1.49 on a scale of 1 to 6), it is possible that the expected negative effect of these problems was not detectable. In addition, the link between externalizing problems and relationship quality with parents may be limited to adolescence. Bivariate correlations show significant links between these problems and conflicts only at ages 16 and 17.

Family cohesion did not predict changes in conflict, and depressive symptoms did not predict changes in intimacy. This lack of association suggests that relationship quality’s positive and negative dimensions are shaped by distinct variables. Thus, the beneficial effect of cohesion appears to be specific to intimacy and is not accompanied by fewer conflicts, and the harmful effect of depressive symptoms seems specific to conflicts. These results highlight the importance of separately examining the positive and negative dimensions of relationship quality.

### Strengths, Limitations, and Future Directions

To our knowledge, this study is the first to examine the development of the positive and negative dimensions of the relationship quality with mothers and fathers separately from adolescence to the end of EA, using many measurement points. Analyzing intimacy and conflict as distinct dimensions and examining them separately for mothers and fathers offered valuable insight into their parent-specific and dimension-specific precursors. These distinctions are important, as they enable the identification of specific factors that promote intimacy or reduce the risk of conflicts with each parent. The interdependence between parents was also considered by exploring with joint trajectories how intimacy and conflicts evolve for each parent in connection with the other. This study therefore provides nuanced results by distinguishing between parents, while considering the links that may exist between them. Furthermore, the analyses conducted allowed the identification of change patterns reported by the majority and subgroups experiencing different pathways. Several studies overlooked this diversity by oversampling college or university students (Oliveira et al., 2020). Initiating this longitudinal study with a sample recruited at a younger age increased the likelihood of including participants with diverse life paths. Accounting for this heterogeneity is essential since our findings indicate that, although parent-child relationships are generally positive during EA, some individuals experience low intimacy and high conflict.

Some limitations must be noted. Intimacy and conflicts with mothers and fathers were measured only from the child’s perspective despite these being dyadic relationships. Using both parent and child perspectives may provide a better understanding of family relationships. All study variables (except socioeconomic status) were based on child reports, which could lead to shared method variance issues. In addition, the sample was culturally and ethnically homogeneous, as it came from a limited region in Quebec. Finally, excluded participants were more likely to come from separated and low-income families, reducing diversity within the sample.

Other characteristics of relationships with mothers and fathers should be examined, such as the different forms of support provided by parents, parental control, and autonomy support (Padilla-Walker & Nelson, 2019). Moreover, a combined examination of intimacy and conflict, using methods such as latent transition analysis, might also be useful for identifying typologies that integrate both dimensions. While this approach would not clarify their respective development and predictors, it would capture individuals’ experiences by considering intimacy and conflict together. This method would also allow for potential links between these two variables to be taken into account. In addition, other predictors of trajectories should be considered, such as transitions experienced by parents and young adults, as they may contribute to changing the parent-child relationship (Lindell, Campione-Barr, 2017). Certain parental characteristics, such as their depressive symptoms, could also be associated with how intimacy and conflicts with their children evolve during this period. Finally, does belonging to the different intimacy and conflict trajectories identified in this study contribute to or hinder adjustment in adulthood? Some studies report links between the quality of these relationships during EA and later adjustment, but few examine the positive aspects of adaptation (e.g., Doty & Mortimer, 2018).

## Conclusion

Findings indicate that intimacy with mothers and fathers tends to increase, and conflicts tend to decrease for most individuals between ages 16 and 30. However, subgroups maintain less intimate and/or more conflictual relationships with their parents. Differences and similarities in relationships with mothers and fathers also support the importance of studying them separately. Spillover and compensation between mother–child and father–child relationships suggest that complex processes are involved in how these two relationships are linked. Finally, examining certain predictors can help understand how to foster positive developments in parent-child relationships during EA. For instance, the findings could raise awareness among practitioners and parents about factors that seem to weaken or strengthen mother–child and father–child relationships during EA, such as depressive symptoms and family cohesion.

## Supplemental Material

sj-docx-1-jbd-10.1177_01650254251364822 – Supplemental material for Trajectories of intimacy and conflict with mothers and fathers from adolescence to adulthood Supplemental material, sj-docx-1-jbd-10.1177_01650254251364822 for Trajectories of intimacy and conflict with mothers and fathers from adolescence to adulthood by Chloé Charest-St-Onge and François Poulin in International Journal of Behavioral Development
