# Supplementary material for: Trajectories of intimacy and conflict with mothers and fathers from adolescence to adulthood
Source: Int J Behav Dev. 2025 Aug 28;50(5):619–31. doi: 10.1177/01650254251364822 (PMC13561413; doi:10.1177/01650254251364822)
Supplement: Supplementary file 1 [file sj-docx-1-jbd-10.1177_01650254251364822.docx]

**Supplemental Material 1: Tables**

**Table 1**

|  |  |  | **Predictors** | | | | | | |
| --- | --- | --- | --- | --- | --- | --- | --- | --- | --- |
|  | Sample size | Mean (Standard Deviation) | Gender | Externalizing Problems | Internalizing Problems | Socioeconomic Status | Family Structure | Family Cohesion |  |
| **Mother intimacy** |  |  |  |  |  |  |  |  |  |
| Age 16 | 291 | 2.87 (1.21) | -.12* | .02 | -.03 | -.14* | -.03 | .21** |  |
| Age 17 | 300 | 2.95 (1.22) | -.19** | -.02 | -.08 | -.12 | -.001 | .24** |  |
| Age 18 | 307 | 3.00 (1.24) | -.19** | -.02 | -.02 | -.18** | .07 | .20** |  |
| Age 19 | 317 | 3.07 (1.20) | -.20** | .02 | .01 | -.14* | .004 | .16** |  |
| Age 20 | 298 | 3.14 (1.24) | -.20** | .01 | -.02 | -.17** | .01 | .12* |  |
| Age 21 | 304 | 3.24 (1.26) | -.14* | .03 | -.02 | -.16* | -.04 | .03 |  |
| Age 22 | 300 | 3.25 (1.25) | -.20** | -.03 | -.01 | -.10 | -.01 | .08 |  |
| Age 25 | 317 | 3.17 (1.22) | -.16** | -.03 | -.06 | -.22** | .003 | .14* |  |
| Age 30 | 316 | 2.89 (1.17) | -.22** | .01 | -.04 | -.07 | -.04 | .11 |  |
| **Father intimacy** |  |  |  |  |  |  |  |  |  |
| Age 16 | 282 | 2.21 (0.98) | .19** | .04 | -.07 | -.08 | .05 | .21** |  |
| Age 17 | 288 | 2.24 (1.00) | .15** | -.02 | -.08 | -.13 | -.02 | .18** |  |
| Age 18 | 293 | 2.31 (1.09) | .001 | .05 | -.06 | -.12 | .02 | .14* |  |
| Age 19 | 305 | 2.40 (1.10) | .12* | .13* | -.02 | -.13* | .01 | .12* |  |
| Age 20 | 290 | 2.49 (1.14) | .08 | .02 | -.11 | -.10 | .06 | .10 |  |
| Age 21 | 291 | 2.53 (1.17) | .10 | .04 | -.03 | -.07 | -.03 | .02 |  |
| Age 22 | 288 | 2.53 (1.10) | .14* | .06 | -.05 | .02 | -.03 | .04 |  |
| Age 25 | 304 | 2.50 (1.21) | .05 | .04 | -.05 | .002 | -.18** | .09 |  |
| Age 30 | 306 | 2.21 (1.02) | .08 | .06 | -.12* | .18** | -.16* | .01 |  |
| **Mother conflict** |  |  |  |  |  |  |  |  |  |
| Age 16 | 291 | 2.04 (0.93) | -.14* | .19** | .30** | .13 | -.003 | -.35** |  |
| Age 17 | 300 | 1.96 (0.86) | -.09 | .17** | .23** | .14* | .02 | -.32** |  |
| Age 18 | 307 | 1.84 (0.88) | -.12* | .07 | .15* | .12 | -.08 | -.17** |  |
| Age 19 | 317 | 1.80 (0.90) | -.10 | -.03 | .21** | .13* | .02 | -.12* |  |
| Age 20 | 298 | 1.65 (0.75) | -.01 | -.08 | .05 | .16* | -.05 | -.04 |  |
| Age 21 | 304 | 1.63 (0.74) | -.11* | -.09 | .11 | .02 | -.001 | -.09 |  |
| Age 22 | 300 | 1.53 (0.70) | -.03 | -.01 | .15** | .13 | -.01 | -.07 |  |
| Age 25 | 317 | 1.49 (0.70) | -.11* | -.01 | .20** | .03 | -.04 | -.11* |  |
| Age 30 | 316 | 1.35 (0.62) | -.13* | .01 | .19** | .04 | .07 | -.11 |  |
| **Father conflict** |  |  |  |  |  |  |  |  |  |
| Age 16 | 282 | 1.98 (0.89) | -.13* | .13* | .20** | .06 | .02 | -.27** |  |
| Age 17 | 288 | 1.91 (0.85) | -.10 | .01 | .09 | .11 | -.13* | -.18** |  |
| Age 18 | 293 | 1.82 (0.82) | .01 | .04 | .10 | .05 | -.01 | -.14* |  |
| Age 19 | 305 | 1.74 (0.81) | -.03 | .02 | .13* | .05 | .06 | -.03 |  |
| Age 20 | 290 | 1.63 (0.75) | -.02 | -.02 | .03 | .09 | -.03 | -.01 |  |
| Age 21 | 291 | 1.55 (0.69) | -.10 | -.10 | .09 | -.04 | .02 | -.05 |  |
| Age 22 | 288 | 1.53 (0.68) | -.01 | .02 | .13* | -.08 | -.04 | -.07 |  |
| Age 25 | 304 | 1.48 (0.69) | -.02 | -.03 | .13* | -.17* | .09 | -.01 |  |
| Age 30 | 306 | 1.36 (0.72) | -.10 | -.04 | .02 | -.17** | .09 | .09 |  |

*Correlations Between Relationship Quality Variables and Predictors*

*Note*. For gender, Female is coded as 0, and Male is coded as 1. For the family structure variable, parents together are coded as 1, and parents separated as 2.

*** = *p*<.001, ** = *p*<.01, * = *p*<.05

**Table 2**

| **Curve shape** | **RMSEA (IC)** | **CFI** | **TLI** | **SRMR** | **Intercept mean** | **Linear slope mean** | **Quadratic slope mean** |
| --- | --- | --- | --- | --- | --- | --- | --- |
| **Mother intimacy** |  |  |  |  |  |  |  |
| Intercept (i) | .121 (.107-.134) | .822 | .851 | .096 | 3.035*** | - | - |
| Linear slope (s) | .111 (.097-.126) | .859 | .873 | .075 | 3.015*** | 0.022 | - |
| Quadratic slope (q) | .051 (.033-.068) | .974 | .974 | .051 | 2.803*** | 1.091*** | -0.749*** |
| **Father intimacy** |  |  |  |  |  |  |  |
| Intercept (i) | .118 (.105-.132) | .771 | .808 | .129 | 2.362*** | - | - |
| Linear slope (s) | .112 (.098-.127) | .809 | .828 | .114 | 2.349*** | 0.009 | - |
| Quadratic slope (q) | .041 (.020-.060) | .976 | .976 | .053 | 2.162*** | 0.967*** | -0.666*** |
| **Mother conflict** |  |  |  |  |  |  |  |
| Intercept (i) | .137 (.124-.151) | .568 | .638 | .168 | 1.667*** | - | - |
| Linear slope (s) | .08 (.066-.095) | .862 | .876 | .087 | 1.926*** | -0.448*** | - |
| Quadratic slope (q) | .050 (.032-.068) | .952 | .952 | .063 | 2.056*** | -1.026*** | 0.381*** |
| **Father conflict** |  |  |  |  |  |  |  |
| Intercept (i) | .127 (.114-.141) | .506 | .587 | .167 | 1.631*** | - | - |
| Linear slope (s) | .072 (.057-.087) | .854 | .868 | .093 | 1.853*** | -0.391*** | - |
| Quadratic slope (q) | .038 (.015-.058) | .962 | .962 | .074 | 1.990*** | -0.987*** | 0.390*** |

*Results of the Latent Growth Model (LGM) Analyses*

*Note*. N for mothers = 368. N for fathers = 361.

*** = *p*<.001, ** = *p*<.01, * = *p*<.05

**Table 3**

*Results of the Latent Class Growth Analyses (LCGA)*

| **Number of classes** | **AIC** | **BIC** | **SSABIC** | **% of the sample by classes**¹ | **Entropy** | **Posterior probabilities**² | **LMR LRT** | **BLRT** |
| --- | --- | --- | --- | --- | --- | --- | --- | --- |
| **Mother intimacy** |  |  |  |  |  |  |  |  |
| 1 | 7323.157 | 7393.502 | 7336.395 | - | - | - | - | - |
| 2 | 7813.764 | 7876.293 | 7825.531 | 43-57 | .869 | .945 | 1074.640*** | 1120.113*** |
| 3 | 7461.526 | 7539.687 | 7476.235 | 29-41 | .847 | .906 | 345.614*** | 360.238*** |
| **3 (q, q, i)** | **7459.803** | **7530.149** | **7473.041** | **30-40** | **.846** | **.910** | **330.030***** | **357.961***** |
| 4 | 7388.744 | 7482.538 | 7406.395 | 14-35 | .81 | .833 | 77.502* | 80.782*** |
| 5 | 7328.469 | 7437.895 | 7349.061 | 5-30 | .807 | .831 | 65.503* | 68.275*** |
| **Father intimacy** |  |  |  |  |  |  |  |  |
| 1 | 6756.632 | 6826.632 | 6769.526 | - | - | - | - | - |
| 2 | 7147.326 | 7209.548 | 7158.788 | 41-59 | .826 | .945 | 804.975*** | 839.149*** |
| 3 | 6888.702 | 6966.479 | 6903.029 | 12-44 | .841 | .907 | 255.767*** | 266.625*** |
| **3 (q, q, i)** | **6885.236** | **6955.236** | **6898.130** | **12-44** | **.841** | **.904** | **245.266***** | **266.090***** |
| 4 | 6828.108 | 6921.441 | 6845.300 | 10-25 | .762 | .792 | 65.801 | 68.594*** |
| 5 | 6788.265 | 6897.154 | 6808.323 | 5-35 | .773 | .807 | 45.894 | 47.842*** |
| **Mother conflict** |  |  |  |  |  |  |  |  |
| 1 | 5446.512 | 5516.858 | 5459.750 | - | - | - | - | - |
| 2 | 5638.942 | 5701.471 | 5650.709 | 19-81 | .918 | .960 | 796.874*** | 830.593*** |
| **2 (s, q)** | **5638.587** | **5697.208** | **5649.619** | **18-82** | **.919** | **.957** | **784.677***** | **828.948***** |
| 3 | 5458.935 | 5537.096 | 5473.644 | 2-76 | .913 | .902 | 180.375 | 188.007*** |
| 4 | 5336.921 | 5430.715 | 5354.572 | 2-51 | .805 | .824 | 124.736* | 130.014*** |
| 5 | 5223.997 | 5333.423 | 5244.589 | 1-51 | .835 | .841 | 116.015* | 120.924*** |
| **Father conflict** |  |  |  |  |  |  |  |  |
| 1 | 5328.044 | 5398.044 | 5340.939 | - | - | - | - | - |
| 2 | 5526.490 | 5588.712 | 5537.952 | 23-77 | .845 | .902 | 564.341 | 588.299*** |
| **2 (q, i)** | **5579.102** | **5633.546** | **5589.131** | **15-85** | **.888** | **.938** | **490.077***** | **531.687***** |
| 3 | 5283.588 | 5361.365 | 5297.915 | 4-71 | .889 | .914 | 240.684* | 250.902*** |
| 4 | 5155.615 | 5248.948 | 5172.808 | 3-67 | .876 | .892 | 130.435 | 135.972*** |
| 5 | 5077.316 | 5186.205 | 5097.374 | 3-65 | .851 | .808 | 82.785 | 86.299*** |

*Note*. N for mothers = 368. N for fathers = 361. 1 = based on membership, 2 = column

*** = *p*<.001, ** = *p*<.01, * = *p*<.05

**Supplemental Material 2: Exploratory analyses**

To explore further the links between the developmental trajectories of mothers and fathers, Parallel-process latent class growth analyses (LCGA) were performed (also named dual or joint trajectory by some authors, even if it differs from the joint trajectories analyses presented in the main manuscript). Parallel-process LCGA is a multivariate extension of univariate LCGA. It is a person-centred approach used to determine classes of individuals with similar developmental patterns for two variables (mother-intimacy and father-intimacy / mother-conflict and father-conflict). Thus, each class identified presents a pattern of changes for intimacy with mothers and for intimacy with fathers (or for conflict with mothers and for conflict with fathers). This method has been used to examine changes in distinct but related variables (e.g., Edgerton et al., 2018; Lee & Ju, 2021; Li et al., 2023; Zhou et al., 2022).

The same set of analyses was carried out first for intimacy and second for conflict. First, a parallel-process latent growth model (LGM) was conducted to determine the form of the growth curve for the entire sample (e.g., linear vs. quadratic) and to verify if the variances estimated for all latent factors (i, s, and q) were significant. Results are presented in Table 4. Second, parallel-process LCGA were conducted to examine the co-development of mother intimacy (or conflict) and father intimacy (or conflict). The same criteria as those described in the main manuscript were used to determine the optimal number of classes (BIC, SSABIC, AIC, BLRT, LMR-LRT, entropy, posterior probability and the number of individuals per class). Results of LCGAs are presented in Table 5 and figures illustrating optimal classes are shown in Figure 1 for intimacy and Figure 2 for conflict.

**Table 4**

| **Curve shape** | **RMSEA (IC)** | **CFI** | **TLI** | **SRMR** |  | **Intercept mean** | **Linear slope mean** | **Quadratic slope mean** |
| --- | --- | --- | --- | --- | --- | --- | --- | --- |
| **Intimacy** |  |  |  |  |  |  |  |  |
| Intercept (i) | .126 (.119-.133) | .667 | .693 | .111 | Mother | 3.034*** | - | - |
|  |  |  |  |  | Father | 2.364*** | - | - |
| Linear slope (s) | .121 (.114-.129) | .708 | .716 | .098 | Mother | 3.010*** | 0.039 | - |
|  |  |  |  |  | Father | 2.343*** | 0.034 | - |
| **Quadratic slope (q)** | **.095 (.088-.103)** | **.836** | **.826** | **.060** | **Mother** | **2.804***** | **1.080***** | **-0.740***** |
|  |  |  |  |  | **Father** | **2.159***** | **0.998***** | **-0.684***** |
| **Conflict** |  |  |  |  |  |  |  |  |
| Intercept (i) | .116 (.109-.123) | .493 | .532 | .141 | Mother | 1.667*** | - | - |
|  |  |  |  |  | Father | 1.632*** | - | - |
| Linear slope (s) | .082 (.074-.089) | .761 | .767 | .092 | Mother | 1.928*** | -0.452*** | - |
|  |  |  |  |  | Father | 1.854*** | -0.391*** | - |
| **Quadratic slope (q)** | **.064 (.055-.072)** | **.867** | **.859** | **.071** | **Mother** | **2.055***** | **-1.014***** | **0.373***** |
|  |  |  |  |  | **Father** | **1.990***** | **-0.988***** | **0.391***** |

*Results of the Parallel-Process Latent Growth Model (LGM) Analyses*

*Note*. N = 361.

*** = *p*<.001, ** = *p*<.01, * = *p*<.05

**Table 5**

*Results of the Parallel-Process Latent Class Growth Analyses (LCGA)*

| **Number of classes** | **AIC** | **BIC** | **SSABIC** | **% of the sample by classes**¹ | **Entropy** | **Posterior probabilities**² | **LMR LRT** | **BLRT** |
| --- | --- | --- | --- | --- | --- | --- | --- | --- |
| **Mother and Father intimacy** |  |  |  |  |  |  |  |  |
| 1 | 13915.444 | 14091.307 | 13948.539 | - | - | - | - | - |
| 2 | 15601.821 | 15722.972 | 15624.620 | 46-54 | .862 | .956 | 1285.449 | 1316.531*** |
| 3 | 15064.339 | 15212.846 | 15092.286 | 24-41 | .881 | .931 | 538.462** | 551.482*** |
| **4** | **14707.451** | **14883.315** | **14740.546** | **11-37** | **.874** | **.900** | **362.132*** | **370.889***** |
| 5 | 14536.863 | 14740.083 | 14575.106 | 10-26 | .852 | .851 | 180.230 | 184.588*** |
| **Mother and Father conflict** |  |  |  |  |  |  |  |  |
| 1 | 10695.479 | 10871.343 | 10728.574 | - | - | - | - | - |
| **2** | **11679.164** | **11800.314** | **11701.963** | **21-79** | **.911** | **.960** | **881.838**** | **903.160***** |
| 3 | 11198.897 | 11347.404 | 11226.844 | 15-65 | .887 | .905 | 482.598 | 494.267*** |
| 4 | 10954.889 | 11130.752 | 10987.984 | 4-60 | .901 | .915 | 251.917 | 258.008*** |
| 5 | 10794.882 | 10998.102 | 10833.125 | 2-58 | .906 | .917 | 169.899 | 174.007*** |

*Note*. N = 361. 1 = based on membership, 2 = column

*** = *p*<.001, ** = *p*<.01, * = *p*<.05

**Figure 1**

*Trajectories of Mothers and Fathers Intimacy from Ages 16 to 30*

*Note.* Age is on the x-axis. Intimacy is on the y-axis. N = 361.

**Figure 2**

*Trajectories of Mothers and Fathers Conflict from Ages 16 to 30*

*Note.* Age is on the x-axis. Conflict is on the y-axis. N = 361.

Similar trajectories to those based on univariate analyses presented in the main manuscript were observed for intimacy and conflict. In fact, for mothers and fathers, trajectories of high quadratic, moderate quadratic and low stable intimacy were distributed across the four co-development classes identified by parallel-process. Moreover, trajectories of high linear decreasing conflict for mothers, higher stable conflict for fathers and low quadratic for both parents were distributed across the two co-development classes identified by parallel-process. This may add confidence to the univariate results presented in the article, as the patterns of change identified were consistent across the different analysis strategies.

It is possible to compare these results with the findings obtained from the joint trajectories based on univariate LCGAs presented in Table 1 and 2 of the main manuscript. Among the nine possible mother/father combinations presented in Table 1 for intimacy, four combinations were observed with parallel-process LCGA: 1) class 1; low stable intimacy for both parents (38% of the sample), 2) class 2; moderate quadratic for both parents (27%), 3) class 3; high quadratic for the mother and low for the father (24%) and 4) class 4; high quadratic for both parents (11%). Among the four possible mother/father combinations presented in Table 2 for conflict, two combinations were observed with parallel-process LCGA: 1) low quadratic for both parents (79%) and 2) higher conflicts with linear decrease for the mother and high stable for the father (21%). This suggests that these classes represent the most salient mother-father combinations of intimacy and conflict, which corresponds in some part to the probabilities presented in Tables 1 and 2. For instance, when an individual reported low or moderate intimacy with one parent, they were likely to be classified in a low-intimacy or a moderate-intimacy group respectively with the other parent as well (with a probability ranging from 55% to 76%). The parallel-process LCGA classes with the most individuals match these joint trajectory results, with 38% of individuals reporting low and stable intimacy with both parents and 27% reporting moderate intimacy with both. For conflicts, Table 2 in the main manuscript indicates a high probability of reporting low conflicts with both parents (probability ranging from 85% to 87%). Accordingly, this is a scenario identified by the parallel-process LCGA in which most of the participants were classified (79% of the sample). However, no class characterized by high conflict with one parent and low conflict with the other was identified, despite de relatively high likelihood (61% to 65% probability) of showing low conflict with one parent when high conflict is present with the other. It is possible that this scenario was not detected by parallel-process LCGA because few individuals in the current sample reported high levels of conflict. This may reflect a less common scenario, which would be more likely to be identified in a larger sample.

In summary, these exploratory analyses were useful in providing a different approach to the co-development of mother-child and father-child relationships during EA. It provided additional information on the most salient mother-father combinations of intimacy and conflict changes across EA. Moreover, the results are consistent with those presented in the article, which may give confidence in the analyses presented in the main manuscript.

# **References**

Edgerton, J. D., Keough, M. T., & Roberts, L. W. (2018). Co-development of Problem Gambling and Depression Symptoms in Emerging Adults : A Parallel-Process Latent Class Growth Model. *Journal of Gambling Studies*, *34*(3), 949‑968. <https://doi.org/10.1007/s10899-018-9760-4>

Lee, Y., & Ju, U. (2021). Joint trajectories of extrinsic motivation and competence beliefs : A parallel-process growth mixture modeling approach. *Contemporary Educational Psychology*, *65*. <https://doi.org/10.1016/j.cedpsych.2021.101944>

Li, Z., Cai, Z., & Yip, P. S. F. (2023). One stream, two channels? A parallel-process latent class growth model of homicide rates and suicide rates in 183 countries, between 2000 and 2019. *SSM - Population Health*, *22*. <https://doi.org/10.1016/j.ssmph.2023.101376>

Zhou, Y., Zheng, H., Liang, Y., Wang, J., Han, R., & Liu, Z. (2022). Joint Developmental Trajectories of Bullying and Victimization from Childhood to Adolescence : A Parallel-Process Latent Class Growth Analysis. *Journal of Interpersonal Violence*, *37*(3‑4), 1759‑1783. <https://doi.org/10.1177/0886260520933054>
